# Correction: Weigelt et al. Too Committed to Switch Off—Capturing and Organizing the Full Range of Work-Related Rumination from Detachment to Overcommitment. *Int. J. Environ. Res. Public Health* 2023, *20*, 3573

**DOI:** 10.3390/ijerph21020203

**Published:** 2024-02-09

**Authors:** Oliver Weigelt, J. Charlotte Seidel, Lucy Erber, Johannes Wendsche, Yasemin Z. Varol, Gerald M. Weiher, Petra Gierer, Claudia Sciannimanica, Richard Janzen, Christine J. Syrek

**Affiliations:** 1 Wilhelm Wundt Institute of Psychology, Leipzig University, D-04109 Leipzig, Germany; 2 Federal Institute for Occupational Safety and Health, Section 3 Work and Health, D-01099 Dresden, Germany; 3 Educational Psychology, Goethe University Frankfurt, D-60629 Frankfurt, Germany; 4 Work and Organizational Psychology, University of Hagen, D-58084 Hagen, Germany; 5 Business Psychology, University of Applied Sciences Bonn-Rhein-Sieg, D-53359 Rheinbach, Germany

In this document we provide a correction to mistakes in [1].

## Error in Tables

1. In the original publication, there was a mistake in Tables 1 and 4 as published. There was a mistake in the calculation of the composite score for overcommitment. The composite score is formed by averaging all the items of the scale. More specifically, in the case of overcommitment, the reverse-scored item was not recoded prior to the calculation of the composite score. Hence, the overcommitment score was underestimated The corrected Table 1 and Table 4 appears below.

2. In the original publication, there was a mistake in Table 7 as published. There was a mistake in the calculation of the composite score for overcommitment. The composite score is formed by averaging all the items of the scale. More specifically, in the case of overcommitment, the reverse-scored item was not recoded prior to the calculation of the composite score. Hence, the overcommitment score was underestimated. This mistake affects the squared correlations between overcommitment and the other constructs above the diagonal only. The average variance extracted and the correlations below the diagonal were estimated based on the item-level data accounting for the polarity of items (whether reverse-scored or not). The corrected Table 7 appears below.

3. In the original publication, there were mistakes in Tables 8–13 as published. In the relative weight analyses, the composite score of overcommitment was entered as a predictor in addition to the other facets of work-related rumination. There was a mistake in the calculation of the composite score for overcommitment. The composite score is formed by averaging all the items of the scale. More specifically, in the case of overcommitment, the reverse-scored item was not recoded prior to the calculation of the composite score. Hence, the overcommitment score was underestimated. The corrected Table 8, Table 9, Table 10, Table 11, Table 12 and Table 13 appear below.

## Text Correction

1. There was a typographic error in the original publication. In the abstract, we state “Second, we leverage apply confirmatory factor analysis…”. The word leverage is not needed. 

A correction has been made to Abstract

Work-related thoughts during off-job time have been studied extensively in occupational health psychology and related fields. We provide a focused review of the research on overcommitment—a component within the effort–reward imbalance model—and aim to connect this line of research to the most commonly studied aspects of work-related rumination. Drawing on this integrative review, we analyze survey data on ten facets of work-related rumination, namely (1) overcommitment, (2) psychological detachment, (3) affective rumination, (4) problem-solving pondering, (5) positive work reflection, (6) negative work reflection, (7) distraction, (8) cognitive irritation, (9) emotional irritation, and (10) inability to recover. First, we apply exploratory factor analysis to self-reported survey data from 357 employees to calibrate overcommitment items and to position overcommitment within the nomological net of work-related rumination constructs. Second, we apply confirmatory factor analysis to self-reported survey data from 388 employees to provide a more specific test of uniqueness vs. overlap among these constructs. Third, we apply relative weight analyses to assess the unique criterion-related validity of each work-related rumination facet regarding (1) physical fatigue, (2) cognitive fatigue, (3) emotional fatigue, (4) burnout, (5) psychosomatic complaints, and (6) satisfaction with life. Our results suggest that several measures of work-related rumination (e.g., overcommitment and cognitive irritation) can be used interchangeably. Emotional irritation and affective rumination emerge as the strongest unique predictors of fatigue, burnout, psychosomatic complaints, and satisfaction with life. Our study is intended to assist researchers in making informed decisions on selecting scales for their research and paves the way for integrating research on the effort–reward imbalance into work-related rumination. 

2. A correction has been made to Section 2. Material and Methods in Section 2.3.13. Emotional Fatigue. In the sample item of emotional fatigue, we incorrectly referred to feeling mentally rather than emotionally exhausted.

We applied all six items of the emotional fatigue subscale of the Work Fatigue Inventory (WFI-3D) by Frone and Tidwell [69]. Instructions, time frame, and response options were the same as for physical and mental fatigue. A sample item is “How often did you feel emotionally exhausted at the end of the workday?”

3. A few percentages reported in the text need to updated as per the corrected Table 12.

A correction has been made to Section 3.6. Relative Predictive Power of the Ten Work-Related Rumination Constructs (Sample 2). As a result of the updated Tables 8–13, the description of the results in the section starting with “Table 12 presents…” needs to be updated to align with the specific percentages displayed in Table 12. The relevant paragraph should read as follows:

Table 12 presents the results for psychosomatic complaints, for which, the ten work-related rumination constructs explained twenty-four percent of the variance. Emotional irritation, affective rumination, and inability to recover were the strongest predictors. Emotional irritation alone explained approximately seven percent of the variance (a fourth of the variance explained by all predictors). Affective rumination uniquely explained six percent of the variance in psychosomatic complaints. Inability to recover and overcommitment explained, respectively, four and two percent of the unique variance in psychosomatic complaints. Negative work reflection, cognitive irritation, and distraction explained less than two percent of the unique variance in psychosomatic complaints.

The authors state that the scientific conclusions are unaffected. This correction was approved by the Academic Editor. The original publication has also been updated.

## Figures and Tables

**Table 1:** Means, standard deviations, and zero-order correlations among study variables (Sample 1).

	Variable	*M*	*SD*	1	2	3	4	5	6	7	8	9	10	11	12	13	14	15	16
1	Age	32.31	9.64																
2	Gender	-	-	−0.02															
3	Working hours per week	27.89	10.80	0.22 ***	−0.02														
4	Leadership position	-	-	0.15 **	−0.09	0.22 ***													
5	Overcommitment (4 Items)	2.68	0.94	0.16 **	0.16 **	0.23 ***	0.12 *	(0.85)											
6	Overcommitment (5 Items)	2.71	0.87	0.13 *	0.18 **	0.22 ***	0.11 *	0.97 ***	(0.82)										
7	Overcommitment (6 Items)	2.73	0.80	0.14 *	0.20 ***	0.21 ***	0.12 *	0.95 ***	0.98 ***	(0.80)									
8	Psychological Detachment (REQ)	3.28	1.01	−0.09	−0.08	−0.24 ***	−0.14 **	−0.75 ***	−0.72 ***	−0.69 ***	(0.92)								
9	Affective Rumination (WRRQ)	2.47	0.94	−0.02	0.13 *	0.11 *	0.08	0.56 ***	0.58 ***	0.59 ***	−0.48 ***	(0.90)							
10	Problem-Solving Pondering (WRRQ)	2.64	0.84	0.18 **	0.04	0.14 **	0.12 *	0.63 ***	0.63 ***	0.61 ***	−0.59 ***	0.41 ***	(0.83)						
11	Positive Work Reflection	2.70	0.91	0.06	−0.08	−0.09	0.04	0.02	0.02	0.01	−0.08	−0.22 ***	0.26 ***	(0.91)					
12	Negative Work Reflection	2.79	0.94	−0.07	0.07	0.08	−0.03	0.42 ***	0.41 ***	0.42 ***	−0.36 ***	0.52 ***	0.35 ***	−0.01	(0.92)				
13	Cognitive Irritation	2.57	1.03	0.11 *	0.11 *	0.23 ***	0.15 **	0.86 ***	0.85 ***	0.83 ***	−0.79 ***	0.63 ***	0.64 ***	0.00	0.47 ***	(0.91)			
14	Emotional Irritation	2.31	0.93	−0.03	0.10	0.09	0.01	0.41 ***	0.43 ***	0.44 ***	−0.30 ***	0.62 ***	0.23 ***	−0.23 ***	0.38 ***	0.46 ***	(0.91)		
15	Distraction (WRRQ)	3.26	1.00	−0.07	−0.14 **	−0.17 **	−0.04	−0.72 ***	−0.72 ***	−0.71 ***	0.76 ***	−0.55 ***	−0.56 ***	0.02	−0.40 ***	−0.76 ***	−0.36 ***	(0.91)	
16	Inability to Recover (FABA)	2.31	0.97	0.09	0.10	0.19 ***	0.14 **	0.76 ***	0.77 ***	0.78 ***	−0.65 ***	0.65 ***	0.61 ***	−0.07	0.53 ***	0.80 ***	0.47 ***	−0.72 ***	(0.87)

Note. *n* = 357. *M* = mean; *SD* = standard deviation; REQ = recovery experience questionnaire; WRRQ = work-related rumination questionnaire; FABA = faulty attitude and behavior analysis. McDonald’s Omega (ω) is on the diagonal in parentheses. Overcommitment (4 Items) = composite of items 2, 3, 4, and 5. Overcommitment (5 Items) = composite of items 2, 3, 4, 5, and 6. Overcommitment (6 Items) = composite of items 1, 2, 3, 4, 5, and 6. We provide confidence intervals for all correlations in Table S4 in the Supplementary Materials. * *p* < 0.05. ** *p* < 0.01. *** *p* < 0.001.

**Table 4:** Means, standard deviations, and zero-order correlations among study variables.

	Variable	*M*	*SD*	1	2	3	4	5	6	7	8	9	10	11	12	13	14	15	16	17	18	19	20	21	22
1	Age	32.10	9.53																						
2	Gender	-	-	−0.10																					
3	Working hours per week	27.20	12.20	0.37 ***	−0.14																				
4	Leadership position	-	-	0.35 ***	−0.12 **	0.18 ***																			
5	Overcommitment (4 Items)	2.71	0.96	0.11 *	0.17 ***	0.18 ***	0.21 ***	(0.83)																	
6	Overcommitment (5 Items)	2.71	0.90	0.07	0.20 ***	0.15 **	0.19 ***	0.97 ***	(0.81)																
7	Overcommitment (6 Items)	2.73	0.85	0.06	0.21 ***	0.14 **	0.19 ***	0.96 ***	0.98 ***	(0.82)															
8	Psychological Detachment (REQ)	3.26	1.02	−0.06	−0.07	−0.14 **	−0.19 ***	−0.73 ***	−0.70 ***	−0.70 ***	(0.91)														
9	Affective Rumination (WRRQ)	2.52	0.97	−0.02	0.17 ***	0.05	0.07	0.62 ***	0.64 ***	0.65 ***	−0.47 ***	(0.91)													
10	Problem-Solving Pondering (WRRQ)	2.64	0.87	0.16 **	0.10	0.10 *	0.23 ***	0.58 ***	0.58 ***	0.58 ***	−0.50 ***	0.40 ***	(0.83)												
11	Positive Work Reflection	2.68	0.97	0.01	0.03	−0.12 *	−0.03	−0.11 *	−0.12 *	−0.13 *	0.10 *	−0.29 ***	0.16 **	(0.93)											
12	Negative Work Reflection	2.92	0.98	−0.08	0.13 *	0.07	0.02	0.40 ***	0.40 ***	0.41 ***	−0.32 ***	0.45 ***	0.30 ***	−0.09	(0.92)										
13	Cognitive Irritation	2.58	1.09	0.07	0.12 *	0.17 **	0.19 ***	0.85 ***	0.85 ***	0.84 ***	−0.79 ***	0.62 ***	0.62 ***	−0.11 *	0.42 ***	(0.90)									
14	Emotional Irritation	2.32	1.00	−0.11 *	0.09	0.01	0.02	0.41 ***	0.44 ***	0.46 ***	−0.36 ***	0.56 ***	0.25 ***	−0.27 ***	0.35 ***	0.47 ***	(0.91)								
15	Distraction (WRRQ)	3.25	1.04	−0.08	−0.11 *	−0.08	−0.19 ***	−0.74 ***	−0.72 ***	−0.71 ***	0.73 ***	−0.56 ***	−0.53 ***	0.09	−0.39 ***	−0.79 ***	−0.38 ***	(0.90)							
16	Inability to Recover (FABA)	2.38	0.96	0.06	0.21 ***	0.15 **	0.22 ***	0.81 ***	0.82 ***	0.84 **	−0.68 ***	0.71 ***	0.58 ***	−0.17 **	0.49 ***	0.82 ***	0.52 ***	−0.74 ***	(0.87)						
17	Physical Fatigue (WFI)	3.20	0.90	−0.04	0.28 ***	0.04	−0.02	0.32 ***	0.32 ***	0.36 ***	−0.29 ***	0.46 ***	0.15 **	−0.26 ***	0.37 ***	0.33 ***	0.42 ***	−0.28 ***	0.43 ***	(0.94)					
18	Mental Fatigue (WFI)	3.25	0.94	−0.11 *	0.23 ***	0.02	0.00	0.41 ***	0.42 ***	0.45 ***	−0.35 ***	0.48 ***	0.20 ***	−0.24 ***	0.37 ***	0.45 ***	0.49 ***	−0.39 ***	0.51 ***	0.63 ***	(0.95)				
19	Emotional Fatigue (WFI)	2.80	1.08	0.00	0.23 ***	0.08	−0.02	0.35 ***	0.36 ***	0.38 ***	−0.27 ***	0.45 ***	0.19 ***	−0.23 ***	0.31 ***	0.38 ***	0.45 ***	−0.33 ***	0.49 ***	0.50 ***	0.71 ***	(0.96)			
20	Personal Burnout (CBI)	2.77	1.06	−0.09	0.19 ***	0.01	−0.04	0.41 ***	0.42 ***	0.45 ***	−0.37 ***	0.55 ***	0.23 ***	−0.32 ***	0.41 ***	0.44 ***	0.55 ***	−0.40 ***	0.53 ***	0.67 ***	0.68 ***	0.61 ***	(0.93)		
21	Psychosomatic Complaints (SCL-90)	1.89	0.67	−0.16 **	0.24 ***	−0.08	−0.07	0.34 ***	0.35 ***	0.36 ***	−0.24 ***	0.43 ***	0.13 **	−0.14 **	0.26 ***	0.32 ***	0.41 ***	−0.28 ***	0.41 ***	0.53 ***	0.47 ***	0.45 ***	0.55 ***	(0.80)	
22	Satisfaction with Life (SWLS)	4.60	1.24	0.01	0.02	0.02	0.10	−0.02	−0.02	−0.03	0.09	−0.18 ***	0.04	0.29 ***	−0.12 *	−0.08	−0.29 ***	0.14 **	−0.16 **	−0.24 ***	−0.28 ***	−0.28 ***	−0.37 ***	−0.19 ***	(0.88)

Note. *n* = 388. *M* = mean; *SD* = standard deviation; REQ = recovery experience questionnaire; WRRQ = work-related rumination questionnaire; FABA = faulty attitude and behavior analysis; WFI = 3D-work fatigue inventory; CBI = Copenhagen burnout inventory; SCL-90 = symptom checklist 90; SWLS = satisfaction with life scale. McDonald’s Omega (ω) reliabilities are on the diagonal in parentheses. Overcommitment (4 Items) = composite of items 2, 3, 4, and 5. Overcommitment (5 Items) = composite of items 2, 3, 4, 5, and 6. Overcommitment (6 Items) = composite of items 1, 2, 3, 4, 5, and 6. We provide confidence intervals for all correlations in Table S5 in the Supplementary Materials. * *p* < 0.05. ** *p* < 0.01. *** *p* < 0.001.

**Table 7:** Average variance extracted and squared correlations among variables in Sample 2.

	Variable	AVE	1	2	3	4	5	6	7	8	9	10
1	Overcommitment (4 Items)	0.53		0.54	0.38	0.33	0.01	0.16	0.72	0.17	0.54	0.66
2	Psychological Detachment (REQ)	0.71	0.57		0.22	0.25	0.01	0.10	0.63	0.13	0.54	0.46
3	Affective Rumination (WRRS)	0.67	0.34	0.22		0.16	0.08	0.20	0.34	0.32	0.31	0.51
4	Problem-Solving Pondering (WRRS)	0.55	0.26	0.20	0.15		0.03	0.09	0.39	0.06	0.28	0.33
5	Positive Work Reflection	0.76	0.01	0.01	0.06	0.00		0.01	0.01	0.07	0.01	0.03
6	Negative Work Reflection	0.74	0.15	0.11	0.17	0.08	0.00		0.18	0.12	0.15	0.24
7	Cognitive Irritation	0.75	0.91	0.85	0.44	0.38	0.01	0.21		0.22	0.63	0.68
8	Emotional Irritation	0.66	0.12	0.11	0.23	0.04	0.04	0.09	0.21		0.14	0.27
9	Distraction (WRRS)	0.65	0.49	0.55	0.26	0.22	0.01	0.14	0.73	0.10		0.54
10	Inability to Recover (FABA)	0.59	0.55	0.46	0.41	0.24	0.02	0.18	0.77	0.16	0.49	

Note. *n* = 388. AVE = average variance extracted. Overcommitment (4 Items) = composite of items 2, 3, 4, and 5; REQ = recovery experience questionnaire; WRRQ = work-related rumination questionnaire; FABA = faulty attitude and behavior analysis. Below the diagonal, we report the squared correlations of latent covariances as estimated in the confirmatory factor analysis. Above the diagonal we report the squared correlations of observed variables (unweighted composite scores).

**Table 8:** Coefficients of the relative weight analyses predicting physical fatigue (Sample 2).

Criterion	Physical Fatigue									
Predictor	*b*	*SE*	*T*	*p*		RW		CI-L	CI-U	RS-RW (%)
Intercept	2.17	0.47	4.59	0.00	***					
Overcommitment (4 Items)	−0.03	0.08	−0.38	0.70		0.0163		0.0083	0.0265	5.32
Psychological Detachment (REQ)	−0.10	0.07	−1.47	0.14		0.0155		0.0056	0.0314	5.05
Affective Rumination (WRRQ)	0.18	0.06	2.79	0.01	**	0.0594	*	0.0304	0.0971	19.34
Problem-Solving Pondering (WRRQ)	−0.09	0.06	−1.44	0.15		0.0055		0.0021	0.0068	1.81
Positive Work Reflection	−0.09	0.04	−2.08	0.04	*	0.0290	*	0.0068	0.0632	9.45
Negative Work Reflection	0.17	0.05	3.59	0.00	***	0.0547	*	0.0237	0.0983	17.82
Cognitive Irritation	−0.05	0.09	−0.60	0.55		0.0160	*	0.0086	0.0232	5.20
Emotional Irritation	0.15	0.05	3.09	0.00	**	0.0602	*	0.029	0.0994	19.61
Distraction (WRRQ)	0.08	0.07	1.28	0.20		0.0107		0.0056	0.0158	3.48
Inability to Recover (FABA)	0.21	0.09	2.38	0.02	*	0.0396	*	0.0212	0.0615	12.90
Adjusted *R*-squared						0.2882				
*F* [10, 377]						16.47				
*p*						<0.001				

Note. *n* = 388. b = unstandardized regression coefficient; SE = standard error; RW = raw relative weight (within rounding error, raw weights will sum to R-squared); CI-L = lower bound of confidence interval used to test the statistical significance of raw weight; CI-U = upper bound of confidence interval used to test the statistical significance of raw weight; RS-RW (%) = relative weight rescaled as a percentage of predicted variance in the criterion variable attributed to each predictor (within rounding error, rescaled weights sum to 100%). Overcommitment (4 Items) = composite of items 2, 3, 4, and 5; REQ = recovery experience questionnaire; WRRQ = work-related rumination questionnaire; FABA = faulty attitude and behavior analysis. * *p* < 0.05. ** *p* < 0.01. *** *p* < 0.001.

**Table 9:** Coefficients of the relative weight analyses predicting mental fatigue (Sample 2).

Criterion	Mental Fatigue									
Predictor	*b*	*SE*	*T*	*p*		RW		CI-L	CI-U	RS-RW (%)
Intercept	2.00	0.48	4.15	0.00	***					
Overcommitment (4 Items)	−0.03	0.09	−0.38	0.70		0.0267		0.0153	0.0401	7.38
Psychological Detachment (REQ)	0.01	0.07	0.09	0.93		0.0191		0.0100	0.0322	5.27
Affective Rumination (WRRQ)	0.08	0.06	1.25	0.21		0.0483	*	0.0257	0.0751	13.34
Problem-Solving Pondering (WRRQ)	−0.14	0.06	−2.22	0.03	*	0.0082		0.0043	0.0099	2.26
Positive Work Reflection	−0.07	0.05	−1.54	0.13		0.0211		0.004	0.0496	5.84
Negative Work Reflection	0.12	0.05	2.50	0.01	*	0.0407	*	0.0167	0.078	11.23
Cognitive Irritation	0.10	0.09	1.08	0.28		0.0338	*	0.0205	0.0509	9.33
Emotional Irritation	0.22	0.05	4.37	0.00	***	0.0865	*	0.0511	0.1321	23.89
Distraction (WRRQ)	0.01	0.07	0.17	0.86		0.0238		0.0124	0.0413	6.58
Inability to Recover (FABA)	0.26	0.09	2.81	0.01	**	0.0539	*	0.0351	0.076	14.88
Adjusted *R*-squared						0.3447				
*F* [10, 377]						20.83				
*p*						<0.001				

Note. *n* = 388. b = unstandardized regression coefficient; SE = standard error; RW = raw relative weight (within rounding error, raw weights will sum to R-squared); CI-L = lower bound of confidence interval used to test the statistical significance of raw weight; CI-U = upper bound of confidence interval used to test the statistical significance of raw weight; RS-RW (%) = relative weight rescaled as a percentage of predicted variance in the criterion variable attributed to each predictor (within rounding error, rescaled weights sum to 100%). Overcommitment (4 Items) = composite of items 2, 3, 4, and 5; REQ = recovery experience questionnaire; WRRQ = work-related rumination questionnaire; FABA = faulty attitude and behavior analysis. * *p* < 0.05. *** *p* < 0.001.

**Table 10:** Coefficients of the relative weight analyses predicting emotional fatigue (Sample 2).

Criterion	Emotional Fatigue									
Predictor	*b*	*SE*	*T*	*p*		RW		CI-L	CI-U	RS-RW (%)
Intercept	1.12	0.56	1.99	0.05	*					
Overcommitment (4 Items)	−0.09	0.10	−0.92	0.36		0.0205		0.0116	0.0307	5.28
Psychological Detachment (REQ)	0.10	0.08	1.29	0.20		0.0106		0.0056	0.0153	3.30
Affective Rumination (WRRQ)	0.08	0.08	1.09	0.28		0.0463	*	0.0238	0.0768	14.16
Problem-solving Pondering (WRRQ)	−0.07	0.07	−0.99	0.32		0.0076		0.0039	0.0102	2.94
Positive Work Reflection	−0.10	0.05	−1.80	0.07	†	0.0227		0.0045	0.0507	7.52
Negative Work Reflection	0.07	0.06	1.21	0.23		0.0260		0.0087	0.0568	9.16
Cognitive Irritation	−0.03	0.11	−0.29	0.77		0.0245		0.0142	0.0369	7.12
Emotional Irritation	0.24	0.06	4.04	0.00	***	0.0779	*	0.0396	0.1251	27.52
Distraction (WRRQ)	−0.02	0.08	−0.28	0.78		0.0194		0.0096	0.0343	5.76
Inability to Recover (FABA)	0.46	0.11	4.31	0.00	***	0.0642	*	0.0399	0.0918	17.24
Adjusted *R*-squared						0.3013				
*F* [10, 377]						17.43				
*p*						<0.001				

Note. *n* = 388. b = unstandardized regression coefficient; SE = standard error; RW = raw relative weight (within rounding error, raw weights will sum to R-squared); CI-L = lower bound of confidence interval used to test the statistical significance of raw weight; CI-U = upper bound of confidence interval used to test the statistical significance of raw weight; RS-RW (%) = relative weight rescaled as a percentage of predicted variance in the criterion variable attributed to each predictor (within rounding error, rescaled weights sum to 100%). Overcommitment (4 Items) = composite of items 2, 3, 4, and 5; REQ = recovery experience questionnaire; WRRQ = work-related rumination questionnaire; FABA = faulty attitude and behavior analysis. † *p* < 0.10. * *p* < 0.05. *** *p* < 0.001.

**Table 11:** Coefficients of the relative weight analyses predicting burnout (Sample 2).

Criterion	Burnout									
Predictor	*b*	*SE*	*T*	*p*		RW		CI-L	CI-U	RS-RW (%)
Intercept	1.86	0.49	3.76	0.00	***					
Overcommitment (4 Items)	−0.09	0.09	−0.99	0.32		0.0229	*	0.0131	0.0333	5.06
Psychological Detachment (REQ)	−0.08	0.07	−1.13	0.26		0.0217	*	0.01	0.0373	4.80
Affective Rumination (WRRQ)	0.19	0.07	2.87	0.00	**	0.0758		0.044	0.1108	16.77
Problem-Solving Pondering (WRRQ)	−0.04	0.06	−0.07	0.51		0.0088	*	0.0047	0.0131	1.94
Positive Work Reflection	−0.56	0.05	−3.42	0.00	***	0.0483	*	0.0195	0.0846	10.67
Negative Work Reflection	0.15	0.05	3.17	0.00	**	0.0519	*	0.0251	0.0899	11.48
Cognitive Irritation	−0.08	0.09	−0.83	0.41		0.0253	*	0.0157	0.0367	5.59
Emotional Irritation	0.30	0.05	5.79	0.00	***	0.1158	*	0.0705	0.1711	25.61
Distraction (WRRQ)	−0.04	0.07	−0.52	0.60		0.0251	*	0.0121	0.0456	5.55
Inability to Recover (FABA)	0.27	0.09	2.96	0.00	**	0.0566		0.0367	0.0784	12.52
Adjusted *R*-squared						0.4377				
*F* [10, 377]						31.28				
*p*						<0.001				

Note. *n* = 388. b = unstandardized regression coefficient; SE = standard error; RW = raw relative weight (within rounding error, raw weights will sum to R-squared); CI-L = lower bound of confidence interval used to test the statistical significance of raw weight; CI-U = upper bound of confidence interval used to test the statistical significance of raw weight; RS-RW (%) = relative weight rescaled as a percentage of predicted variance in the criterion variable attributed to each predictor (within rounding error, rescaled weights sum to 100%). Overcommitment (4 Items) = composite of items 2, 3, 4, and 5; REQ = recovery experience questionnaire; WRRQ = work-related rumination questionnaire; FABA = faulty attitude and behavior analysis. * *p* < 0.05. ** *p* < 0.01. *** *p* < 0.001.

**Table 12:** Coefficients of the relative weight analyses predicting psychosomatic complaints (Sample 2).

Criterion	Psychosomatic Complaints									
Predictor	*b*	*SE*	*T*	*p*		RW		CI-L	CI-U	RS-RW (%)
Intercept	0.75	0.37	2.06	0.04	*					
Overcommitment (4 Items)	0.06	0.07	0.87	0.38		0.0224		0.0109	0.0378	8.77
Psychological Detachment (REQ)	0.02	0.05	0.46	0.65		0.0088		0.0037	0.0157	3.44
Affective Rumination (WRRQ)	0.15	0.05	3.07	0.00	**	0.0602	*	0.0322	0.0979	23.59
Problem-Solving Pondering (WRRQ)	−0.12	0.05	−2.46	0.01	*	0.0064		0.0027	0.012	2.52
Positive Work Reflection	0.03	0.04	0.97	0.34		0.0039		0.0013	0.0118	1.54
Negative Work Reflection	0.02	0.04	0.47	0.64		0.0153		0.0049	0.0352	5.99
Cognitive Irritation	−0.02	0.07	−0.34	0.73		0.0173		0.0088	0.0285	6.76
Emotional Irritation	0.15	0.04	3.85	0.00	***	0.0680	*	0.0324	0.119	26.64
Distraction (WRRQ)	0.01	0.05	−0.23	0.81		0.0130		0.0061	0.0241	5.08
Inability to Recover (FABA)	0.16	0.07	2.34	0.02	*	0.0400	*	0.0214	0.0635	15.67
*R*-squared						0.2357				
*F* [10, 377]						13.00				
*p*						<0.001				

Note. *n* = 388. b = unstandardized regression coefficient; SE = standard error; RW = raw relative weight (within rounding error, raw weights will sum to R-squared); CI-L = lower bound of confidence interval used to test the statistical significance of raw weight; CI-U = upper bound of confidence interval used to test the statistical significance of raw weight; RS-RW (%) = relative weight rescaled as a percentage of predicted variance in the criterion variable attributed to each predictor (within rounding error, rescaled weights sum to 100%). Overcommitment (4 Items) = composite of items 2, 3, 4, and 5; REQ = recovery experience questionnaire; WRRQ = work-related rumination questionnaire; FABA = faulty attitude and behavior analysis. * *p* < 0.05. ** *p* < 0.01. *** *p* < 0.001.

**Table 13:** Coefficients of the relative weight analyses predicting satisfaction with life (Sample 2).

Criterion	Satisfaction with Life									
Predictor	*b*	*SE*	*T*	*p*		RW		CI-L	CI-U	RS-RW (%)
Intercept	2.68	0.70	3.82	0.00	***					
Overcommitment (4 Items)	0.45	0.13	3.57	0.00	***	0.0145		0.0054	0.0307	7.77
Psychological Detachment (REQ)	0.06	0.10	0.60	0.55		0.0053		0.0016	0.0086	2.85
Affective Rumination (WRRQ)	0.04	0.09	0.39	0.69		0.0102		0.0048	0.0218	5.46
Problem-Solving Pondering (WRRQ)	0.10	0.09	1.16	0.25		0.0078		0.0026	0.0235	4.17
Positive Work Reflection	0.27	0.07	4.03	0.00	***	0.0568	*	0.0237	0.1023	30.39
Negative Work Reflection	−0.03	0.07	−0.45	0.65		0.0055		0.0016	0.0204	2.96
Cognitive Irritation	0.12	0.13	0.89	0.38		0.0076		0.0027	0.0105	4.04
Emotional Irritation	−0.28	0.07	−3.83	0.00	***	0.0496		0.0201	0.0957	26.55
Distraction (WRRQ)	0.22	0.10	2.20	0.03	*	0.0124		0.0038	0.0332	6.66
Inability to Recover (FABA)	−0.35	0.13	−2.68	0.01	**	0.0171		0.007	0.0322	9.16
*R*-squared						0.1655				
*F* [10, 377]						8.72				
*p*						<0.001				

Note. *n* = 388. b = unstandardized regression coefficient; SE = standard error; RW = raw relative weight (within rounding error, raw weights will sum to R-squared); CI-L = lower bound of confidence interval used to test the statistical significance of raw weight; CI-U = upper bound of confidence interval used to test the statistical significance of raw weight; RS-RW (%) = relative weight rescaled as a percentage of predicted variance in the criterion variable attributed to each predictor (within rounding error, rescaled weights sum to 100%). Overcommitment (4 Items) = composite of items 2, 3, 4, and 5; REQ = recovery experience questionnaire; WRRQ = work-related rumination questionnaire; FABA = faulty attitude and behavior analysis. * *p* < 0.05. ** *p* < 0.01. *** *p* < 0.001.
